# Biocontrol Efficiency of Endophytic Fungi Against Stem-Rot in *Cymbidium goeringii*

**DOI:** 10.3390/microorganisms14040758

**Published:** 2026-03-27

**Authors:** Xiaotong Ji, Kaili Zhang, Tiankai Shen, Yanru Duan, Lu Xu, Ye Ai, Yuzhen Zhou, Donghui Peng

**Affiliations:** 1 Key Laboratory of National Forestry and Grassland Administration for Orchid Conservation and Utilization at College of Landscape Architecture and Art, Fujian Agriculture and Forestry University, Fuzhou 350002, China; jixiaotong@fafu.edu.cn (X.J.); zklrt20@163.com (K.Z.); 3236610016@stu.fafu.edu.cn (T.S.); yanruduan@fafu.edu.cn (Y.D.);; 2 Fuzhou Sanjiangkou Botanical Garden, Fuzhou 350028, China

**Keywords:** *Cymbidium goeringii*, root endophytic fungi, growth-promotion, stem-rot resistance

## Abstract

*Cymbidium goeringii*, an important orchid species, holds significant aesthetic and commercial potential in horticulture. However, stem rot caused by *Fusarium oxysporum* has emerged as a major biological constraint hindering industry development. In this study, we isolated five endophytic fungal strains from *C. goeringii* roots—namely, DG3 (*Bjerkandera*), DG4 (*Cylindrocarpon*), CLG3 (*Talaromyces*), CLG6 (*Clonostachys*), and Z3 (*Trichoderma*)—and assessed their inhibitory efficacy against stem rot and their potential to promote growth in *C. goeringii*. In vitro assays indicated that all five fungal strains had the ability to fix nitrogen and produce indole-3-acetic acid, as well as the capability to produce protease and exert broad-spectrum antimicrobial effects. The five endophytic fungal strains exhibited stem rot-resistant effects, among which strain Z3 showed the best inhibitory effect against stem rot, with a control efficacy reaching 68.89%. Treatment of *C. goeringii* seedlings with these endophytic fungal fermentation broths for 100 d significantly promoted growth compared to the control. The fresh weight increased by 10.53% to 88.16%, and root activity was enhanced by 50% to 162.5%. Additionally, the plant height and the longest leaf length increased by up to 23.68% and 47.50%, respectively, compared to the control. Additionally, the total chlorophyll content was up to 25.34% higher than that of the control group, and the soluble protein content was up to 39.54% higher. The MDA content was reduced by up to 40.23% compared to the control group. These endophytes also regulated the activity of defense-related enzymes in *C. goeringii*, including delaying the decline in the activities of antioxidant enzymes such as superoxide dismutase, peroxidase, and catalase. These results highlight the potential of these five endophytic fungi as effective agents for managing stem rot in *C. goeringii*.

## 1. Introduction

*Cymbidium goeringii*, a member of the genus *Cymbidium* (Orchidaceae), is valued for its elegant flowers and delicate fragrance in ornamentation, culture, medicine, and research [1]. However, the expansion of its large-scale cultivation has led to a rise in disease incidence, which has become a major constraint to the industry’s healthy development [2]. Current primary measures for the prevention and control of disease include agricultural control, chemical control, and biological control. Agricultural control achieves disease control through the breeding and selection of disease-resistant varieties, but its drawbacks lie in the long breeding period and high cost [3]. Chemical control, which involves the use of chemical agents, can provide relatively rapid disease control [4]. However, it is prone to issues such as the development of pathogen resistance and environmental pollution following pesticide application. Biological control refers to the use of biocontrol agents or their active metabolites to inhibit pathogens, thereby reducing the occurrence of diseases. It has the advantages of reducing the use of chemical pesticides and achieving ecological protection [5]. The *Aureobasidium* sp. JRF1 strain isolated from tomato can prevent disease occurrence by activating auxin and cytokinin signaling pathways [6]; the *F. oxysporum* strain BEN48 isolated from *Prunus yedoensis* trees had the highest inhibition rates against *Ganoderma gibbosum*, *Trametes versicolor*, and *Vanderbylia fraxinea* [7]; the application of these endophytic fungi provides a practical solution for reducing reliance on conventional chemical fungicides and fertilizers. *C.goeringii* stem rot is one of the main diseases affecting its growth and yield. Its pathogens are diverse, primarily including fungi such as *Fusarium* spp., *Phytophthora* spp., and *Pythium* spp. [8]. Biological control of these diseases can reduce the use of chemical pesticides and achieve sustainable agricultural development, making it a current research hotspot [9,10].

Biological control of stem rot in orchid plants primarily involves the introduction of beneficial microorganisms or their metabolites to inhibit the growth and reproduction of pathogenic fungi and enhance the plant’s own disease resistance [11,12]. The genera *Ceratobasidium* and *Tulasnella*, which are often reported as mutualistic symbionts in orchids, belong to a group of fungi with significant potential for pathogen biocontrol. In a dual culture, Manrique et al. found that they can generate a biocontrol effect against *Fusarium* through the mechanisms of antibiosis and competition for space and nutrients [13]. *Ceratobasidium* strains isolated from roots of Colombian orchids demonstrated biocontrol efficacy against sheath blight [14]. Four *Streptomyces* strains (DR5-1, DR7-3, DR8-5, DR8-8), isolated from *Dendrobium*, demonstrated significant antifungal activity against five phytopathogens, showing particularly high potency against *Curvularia oryzae* [15]. A *F. oxysporum* strain KB-3 isolated from the roots of *Bletilla* striata exhibits orchid mycorrhizal fungi characteristics and can promote the germination of *Bletilla striata* seeds [16]. These studies all indicate that endophytic fungi can play a significant role in the biological control of large-scale cultivation of orchids. However, currently, research on the biological control of *C. goeringii* stem rot disease using endophytic fungi remains quite limited.

In this study, we isolated five endophytic fungal strains from *C. goeringii* roots, and assessed their inhibitory efficacy against stem rot and their potential to promote growth. The evaluation included in vitro and in vivo antimicrobial activity, growth-promoting effects, reactive oxygen species metabolism, and disease defense capacity. This study will provide both a theoretical basis and practical measures for the effective control of stem rot in *C. goeringii*.

## 2. Materials and Methods

### 2.1. Plant Materials

The tissue-cultured seedlings of *C. goeringii* were generated by the Key Laboratory of National Forestry and Grassland Administration for Orchid Conservation and Utilization at College of Landscape Architecture and Art, Fujian Agriculture and Forestry University. The seeds were obtained from artificially cultivated plants in this laboratory. The experimental plants were then obtained by hardening these tissue-cultured seedlings. Healthy seedlings with an average plant height of 18 cm and 8–10 fully expanded leaves were selected and transplanted into a mixed substrate of bark:perlite:peat soil = 3:6:2 (by volume). The seedlings were acclimatized for two weeks in a cultivation environment with 75% humidity, 80 Lm luminous flux, and a temperature of 25 ± 1 °C under a 12 h light/12 h dark photoperiod, after which the formal experiment commenced.

### 2.2. Isolation and Identification of Endophytic Fungi from C. goeringii

Root segments were randomly collected from 10 healthy *C. goeringii* plants, first cleaned with a detergent solution, and then rinsed under running water for 1 h. In an ultra-clean workbench, the cleaned root segments were immersed in a 75% (*v*/*v*) ethanol solution for 30 s, rinsed three times with sterile water, surface-sterilized in a 2% (*v*/*v*) sodium hypochlorite solution for 5 min, and then rinsed five times with sterile water. Three surface sterilized root segments (0.5 cm) were placed with their cut surfaces facing down on each 9 cm diameter PDA Petri dish and incubated at 28 °C with daily observation. Additionally, aliquots of the final rinsing solution were spread onto PDA plates as a control to verify the success of the root surface sterilization. The mycelia growing out from the root segments were transferred to PDA slant culture media. These slants were incubated in the dark at 28 °C for 7 d. After the mycelia had fully covered the slant surface, they were stored at 4 °C for preservation.

The morphological characteristics of each fungal colony were observed and recorded. A small amount of mycelium was mounted on a slide and examined under a microscope at 200× magnification to observe the morphology of the hyphae and spores, allowing for morphological identification. Fungal DNA was extracted using the D5542 SP Fungal Mini Kit according to the manufacturer’s instructions. The ITS region was amplified using the universal primers ITS1 and ITS4, and the PCR products were sequenced by Shanghai Biotechnology Co., Ltd., Shanghai, China [17,18]. For molecular identification, the obtained ITS sequences were compared to known sequences in the NCBI database using the BLAST tool (Version 2.17.0.) Sequences with high homology were downloaded, and multiple sequence alignment and phylogenetic analysis were performed using ClustalX and MEGA 6.0 software, respectively. The phylogenetic tree was constructed with the Neighbor-Joining method (Bootstrap = 1000).

### 2.3. The Co-Cultivation of Endophytic Fungi with Arabidopsis Thaliana

The surface sterilization of *Arabidopsis thaliana* seeds was performed using a gradient sterilization method: the seeds were placed in a 1.5 mL centrifuge tube, first treated with 1.2 mL of 70% ethanol with shaking for 1 min. After removing the ethanol, the seeds were disinfected with 1.2 mL of 4% sodium hypochlorite solution (containing available chlorine) for 3 min, followed by five rinses with sterile water to completely remove residual disinfectants. Finally, 1.0 mL of sterile water was added, and the seeds were vernalized at 4 °C for 72 h. The entire operation was conducted in an ultra-clean workbench to ensure aseptic conditions. The co-cultivation experiment of *Arabidopsis thaliana* seeds was carried out using a standard seeding method: the sterilized and vernalized seeds were evenly sown on the second transverse line of 100 mm square MS medium plates (12 seeds per plate). Simultaneously, three fresh fungal plugs (7 mm diameter) of the tested strains were inoculated on the sixth transverse line on the opposite side of the medium, with PDA medium plugs set as the control. All plates were placed vertically in an incubator and cultured at 21 ± 1 °C under a 14 h/d light photoperiod for 7 d. Three biological replicates were set for each treatment group.

### 2.4. The Ability of Endophytic Fungi to Synthesize IAA and Nitrogen-Fixing Capacity

The ability of the endophytic fungi to synthesize IAA was determined using the Salkowski colorimetric method. Purified strains were prepared into 7 mm fungal discs and inoculated in 50 mL of PDB medium supplemented with 100 mg/L tryptophan. A PDB medium without tryptophan served as the negative control. The cultures were incubated at 28 °C with shaking at 190 rpm for 5 d. Then, 200 μL of the supernatant was mixed with an equal volume of Salkowski’s reagent, using a 50 mg/L IAA standard solution as the positive control. After a 30 min reaction in the dark, the color development was observed. The formation of a pink to red color indicated IAA synthesis, and its intensity was positively correlated with the IAA yield. Absorbances of these solutions were measured at 530 nm using a spectrophotometer. A standard curve was generated using standard IAA solutions (0–100 mg/L), and the IAA concentration in the samples was calculated based on the absorbance values. Each treatment was replicated three times.

The nitrogen-fixing ability of the strains was assessed by inoculating 7 mm fungal discs on Ashby’s medium. The plates were incubated upside down at 28 °C in a constant-temperature incubator. A strain that exhibited growth after five consecutive transfers on this nitrogen-free medium was confirmed as a nitrogen-fixer. Each treatment was replicated three times.

### 2.5. Plate Confrontation Assays

The pathogenic fungal *Colletotrichum truncatum*, *Fusarium solani*, *Fusarium oxysporum*, *Colletotrichum gloeosporioides*, *Monilinia fructicola*, and *Cladosporium fulvum* preserved in our laboratory was used in this experiment. Under sterile conditions, the endophytic and pathogenic fungal strains were first revived on PDA plates. When the colonies reached two-thirds of the Petri dish diameter, mycelial discs (7 mm in diameter) were aseptically obtained from each colony using a cork borer. A disc of the pathogenic fungus was placed at the center of a fresh PDA plate. Four discs of an endophytic fungus were then inoculated around it, each positioned approximately 2 cm away at the top, bottom, left, and right. Plates inoculated with only the pathogenic fungus disc served as the control. All plates were incubated at 28 °C. When the fungal colony in the control group covered two-thirds of the plate, the colony diameter (mm) of both the control and treatment groups was measured using the cross-hair method. Each treatment was replicated three times. The inhibition rate of mycelial growth was calculated as follows: Inhibition rate (%) = [(Diameter of control colony − Diameter of treated colony)/(Diameter of control colony − Diameter of fungal disc)] × 100.

### 2.6. The Effects of Endophytic Fungi on the Growth Status of Tissue-Cultured Seedlings of C. goeringii

Healthy, contamination-free tissue-cultured seedlings were selected after one week of culture on MS medium and used for the inoculation assay. A 7 mm-diameter mycelial disc of the endophytic fungus was placed on the medium surface next to each seedling and gently pressed to ensure partial contact with and embedding in the medium, with one disc per bottle. The experiment included ten replicates per treatment. The control group consisted of seedlings inoculated with a sterile agar disc instead of a mycelial one. Seedling growth parameters, including fresh weight, plant height, length of the longest leaf, number of leaves, and number of roots, were measured both before inoculation and after 30 d of co-culture.

### 2.7. The Antagonistic Effects of Endophytic Fungi Against F. oxysporum in Tissue-Cultured Seedlings of C. goeringii

The resistance of *C. goeringii* tissue-cultured seedlings to *F. oxysporum* was evaluated using a detached leaf assay [19,20]. The in vitro leaf experiment was conducted after the seedlings had been co-cultured with the endophytic fungi for 30 d. The longest healthy leaf from the center of a seedling was selected for the test. A 5 mm-diameter mycelial disc of *F. oxysporum* was inoculated beside the midvein of each leaf. Three pieces of sterilized gauze were placed into each 10 × 10 cm disposable sterile square Petri dish, and 15 mL of sterile distilled water was added until it was fully absorbed by the gauze with no visible standing water. Detached leaves were then placed on the moist gauze, the dishes were sealed, and incubated in a constant temperature incubator at 25 ± 1 °C under a 12 h light/12 h dark photoperiod. After 7 d of incubation, the lesion area was measured. The disease area was selected using the magic wand tool of Photoshop 2024, and the area of the disease in the photographed image was measured. The inhibition rate (%) was calculated as [(lesion area in the control group − lesion area in the treatment group)/lesion area in the control group] × 100%, which was used to evaluate the effect of different treatments on the resistance of *C. goeringii* leaves to *F. oxysporum*. Data were analyzed for significant differences using SPSS 19.0 statistical software.

### 2.8. The Effects of Endophytic Fungi Fermentation Broth on C. goeringii Seedlings

Three mycelial discs (7 mm in diameter) were inoculated into 100 mL of PDB and cultured at 28 °C with shaking at 120 rpm for 7 d. The fermentation broth, after being filtered through eight layers of gauze, was placed into 50 mL centrifuge tubes and centrifuged at 7000 rpm for 10 min at 4 °C. After centrifugation to remove mycelia and spores, the supernatant was collected and sterilized by filtration through a 0.22 μm pore-size membrane filter to obtain a sterile, cell-free fermentation filtrate [21,22]. This filtrate was diluted to a 10% (*v*/*v*) concentration with sterile water. For the treatment group, the roots of each seedling were irrigated with 20 mL of this diluted filtrate once per week for three consecutive weeks, the control group was irrigated with an equal volume of sterile water on the same schedule. After the irrigation treatment, the plants were cultivated in a greenhouse at a temperature of 20–25 °C and a humidity of 60–80%, and were watered thoroughly every 7 d until measurement on day 100. All plants were maintained under identical environmental conditions throughout the experiment. Each treatment consists of 3 biological replicates, with each replicate containing 10 seedlings.

Growth and physiological indicators of *C. goeringii* seedlings were measured before and after the 100 d experimental period. Growth parameters, including fresh weight, plant height, length of the longest leaf, number of leaves, length of the longest root, and number of roots, were recorded. Physiological indicators were assessed after 100 d using specific commercial assay kits from Shanghai Youxuan Biotechnology Co., Ltd., Shanghai, China, as follows: Plant Chlorophyll Content Assay Kit (UPLC-W-C112) for chlorophyll content, Plant BCA Protein Quantification Assay Kit (UPLC-MS-5142) for soluble protein content, Malondialdehyde (MDA) Content Assay Kit (UPLC-W-A401) for MDA content, Superoxide Dismutase (SOD) Activity Assay Kit (UPLC-MS-5048) for SOD activity, Peroxidase (POD) Activity Assay Kit (UPLC-MS-4539) for POD activity, and Catalase (CAT) Activity Assay Kit (UPLC-MS-4533) for CAT activity.

*F. oxysporum* was cultured on PDA medium for 5 d. Mycelial discs (7 mm in diameter) were obtained from the colony margins, and five discs were inoculated into sterilized PDB. The culture was incubated at 28 °C with shaking at 180 rpm for 5 d to promote sporulation. The fungal culture was filtered through three layers of sterile gauze to remove bulk mycelia. The filtrate, containing spores and hyphal fragments, was centrifuged at 12,000× *g* for 3 min at room temperature. The supernatant was discarded, and the pellet was resuspended in sterile water. This washing step was repeated twice. The final spore pellet was resuspended in a known volume of sterile water, and the spore concentration was determined using a hemocytometer. The suspension was then adjusted with sterile water to a final concentration of 1 × 10^6^ CFU/mL to obtain the working spore suspension. A disposable inoculation needle was used to inject 1 mL of the *F. oxysporum* spore suspension at a concentration of 1 × 10^6^ CFU/mL into the pseudobulb of *C. goeringii*. The plants were placed in a cultivation environment with 75% humidity, a luminous flux of 80 Lm, and a temperature of 25 ± 1 °C under a 12 h light/12 h dark cycle. Disease severity was assessed 7 d post-inoculation. Each treatment consists of 3 biological replicates, with each replicate containing 5 seedlings. Disease grading for *C. goeringii* stem rot was performed according to the method of Yuan Lihong et al. (Table 1). The disease index was calculated, and the relative control efficacy was determined. The disease severity scale diagram of stem rot in *C. goeringii* is shown in Figure S1.

Disease index = 100 × ∑(Number of diseased plants at each grade × Representative value of each grade)/(Total number of surveyed plants × Representative value of the highest grade). Relative control efficacy (%) = [(Disease index in control group − Disease index in treatment group)/Disease index in control group] × 100.

### 2.9. Statistical Analysis

All experiments in this study were conducted with at least three biological replicates. The data were analyzed using SPSS 19.0, and Duncan’s Multiple Range Test was used to determine significant differences.

## 3. Results

### 3.1. Isolation and Identification of Endophytic Fungi from C. goeringii

**Figure 1:**
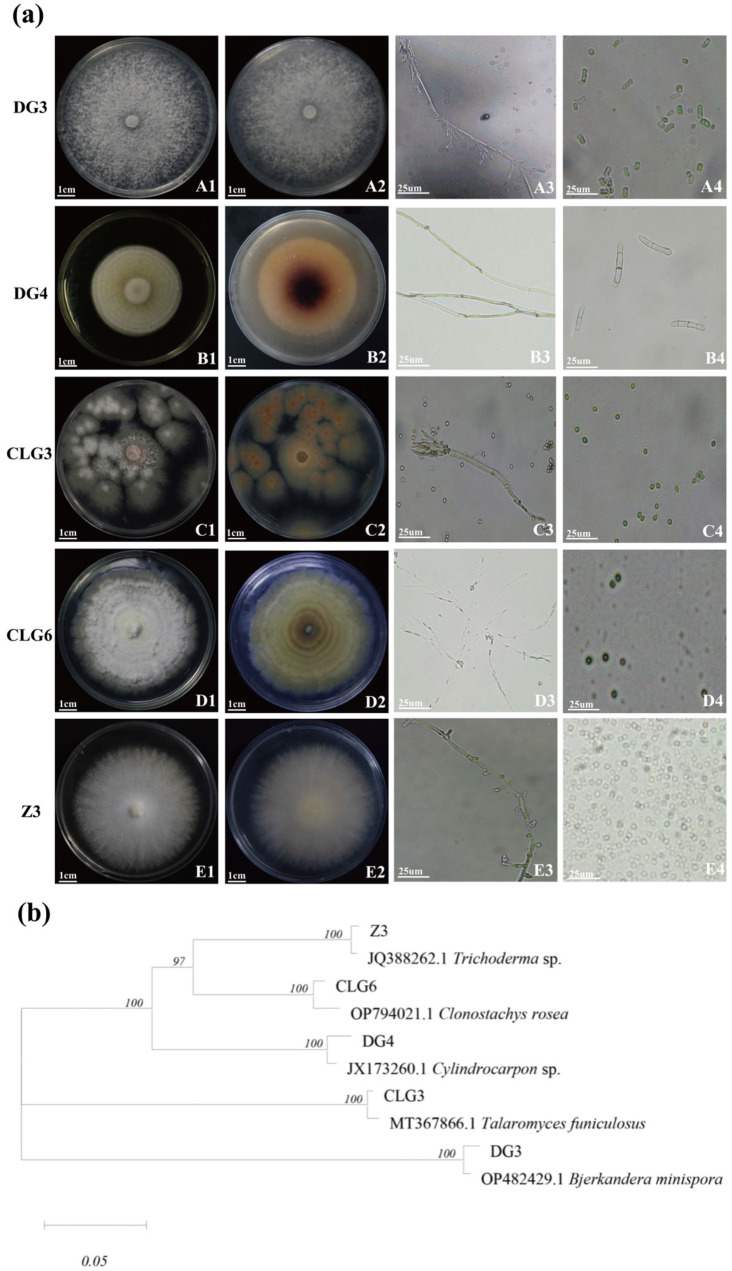
Biological morphological characteristics and phylogenetic tree of five endophytic fungi. (**a**): A1~E1: Front view of colony morphology of fungi DG3, DG4, CLG3, CLG6, Z3; A2~E2: Rear view of colony morphology of fungi DG3, DG4, CLG3, CLG6, Z3; A3~E3: Hyphal morphology of fungi DG3, DG4, CLG3, CLG6, Z3; A4~E4: Spore morphology of fungi DG3, DG4, CLG3, CLG6, Z3. (**b**): rDNA ITS-based phylogenetic tree.

The isolated endophytic fungi were placed on potato dextrose agar (PDA) Petri dishes with a diameter of 9 cm and incubated upside-down at a constant temperature of 28 °C (Figure 1a). The morphological characteristics of the colonies of the five specific endophytic fungi are detailed in Table 2.

The target sequences were analyzed by NCBI BLAST, and highly homologous sequences were used for phylogenetic tree construction. The phylogenetic tree analysis revealed the following relationships: strain DG3 exhibited the closest affinity to *Bjerkandera* (OP482429.1); strain DG4 showed the closest relationship to *Cylindrocarpon* (JX173260.1); strain CLG3 was most closely related to *Talaromyces* (MT367866.1); strain CLG6 demonstrated the nearest phylogenetic ties to *Clonostachys* (OP794021.1); and strain Z3 exhibited the closest relationship to *Trichoderma* (JQ388262.1) (Figure 1b). The base sequences of these five isolated endophytic fungal strains are provided in the Supplementary Table S4.

### 3.2. The Growth-Promoting Potential of Five Endophytic Fungal Strains

**Figure 2:**
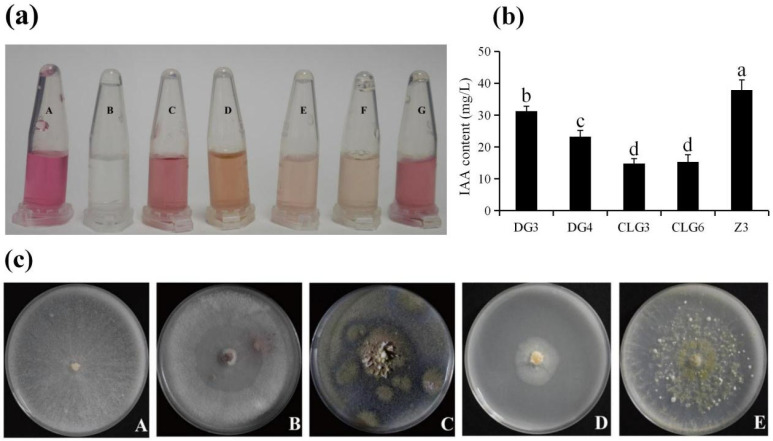
Qualitative analysis of IAA production capability and nitrogen-fixing capability of 5 strains of endophytic fungi. (**a**): A–G were 50 mg/L IAA standard solution, blank group without tryptophan, DG3, DG4, CLG3, CLG6, Z3. (**b**):The IAA-producing capacity of DG3, DG4, CLG3, CLG6, Z3. Data are expressed as mean ± standard deviation (SD). Different lowercase letters indicate significant differences (*p* < 0.05) between different treatments. (**c**): A–E represent the status of DG3, DG4, CLG3, CLG6, Z3 on Ashby’s medium.

Endophytic fungi can promote host plant growth through IAA secretion and tolerance to nitrogen limitation. We detected the IAA synthesis capacity of five endophytic fungal strains using the Salkowski colorimetric method. Positive strains developed a pink color within 30 min, and the color intensity correlated with IAA production levels (Figure 2a). The absorbance of the samples was measured at 530 nm using a spectrophotometer, and the IAA concentration was calculated. The IAA-producing capacity ranked as follows: Z3 > DG3 > DG4 > CLG6 > CLG3 (Figure 2b). The nitrogen-fixing ability of five endophytic fungal strains (DG3, DG4, CLG3, CLG6, and Z3) was assessed. All five strains demonstrated sustained growth after five cyclic spot inoculations on Ashby’s medium, indicating their nitrogen-fixing capability (Figure 2c).

**Figure 3:**
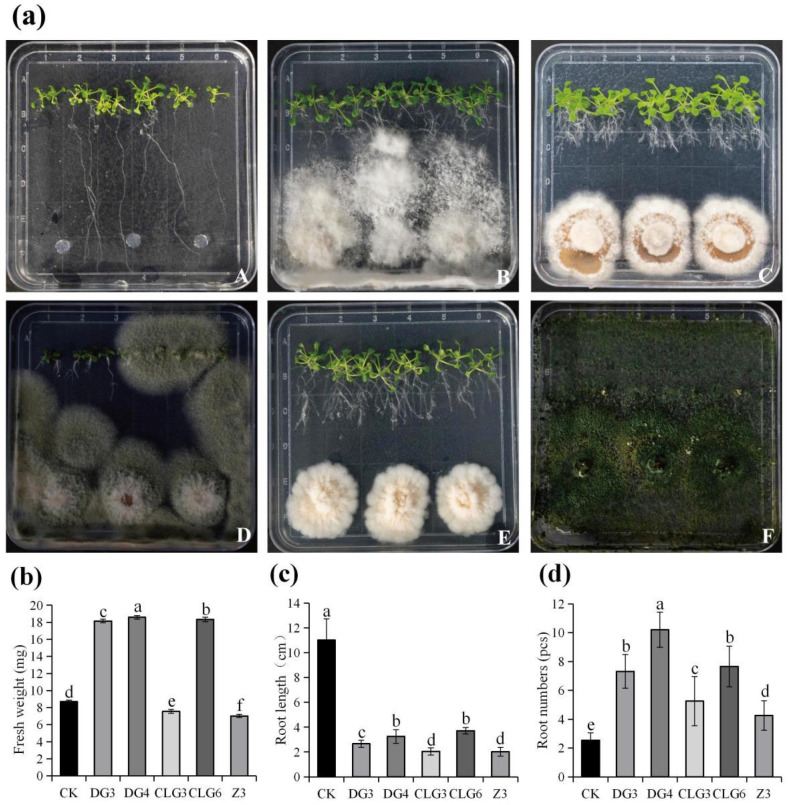
The effect of endophytic fungi on *Arabidopsis thaliana* seed growth. (**a**): The growth condition of *Arabidopsis* (A: CK; B: DG3; C: DG4; D: CLG3; E: CLG6; F: Z3). The effect of endophytic fungi on *Arabidopsis* fresh weight (**b**), root length (**c**), and root numbers (**d**). Data are expressed as mean ± standard deviation (SD) (*n* ≥ 3). Different lowercase letters indicate significant differences (*p* < 0.05) between different treatments.

The effect of endophytic fungi on *Arabidopsis thaliana* seed growth was measured (Figure 3a). After 7 d of co-cultivation, the fresh weight of *Arabidopsis* in the DG3, DG4, and CLG6 treatment groups increased by 108.62%, 113.56%, and 110.69%, respectively, compared to the control group (Figure 3b). Root length decreased by 75.80%, 70.48%, and 66.30%, respectively, relative to the control, while the number of lateral roots increased by 187.80%, 301.97%, and 201.57%, respectively (Figure 3c,d). In summary, strains CLG6, DG3, and DG4 significantly promoted the growth of *Arabidopsis thaliana* seedlings, markedly increasing seedling fresh weight and lateral root number. Strain DG4 showed the strongest ability to enhance both fresh weight and lateral root production. The observed inhibition of root length may be attributed to a growth regulatory response in *Arabidopsis*, whereby increased lateral root formation suppresses primary root elongation.

### 3.3. Antimicrobial Effects of Five Endophytic Fungal Strains In Vitro and In Vivo

**Figure 4:**
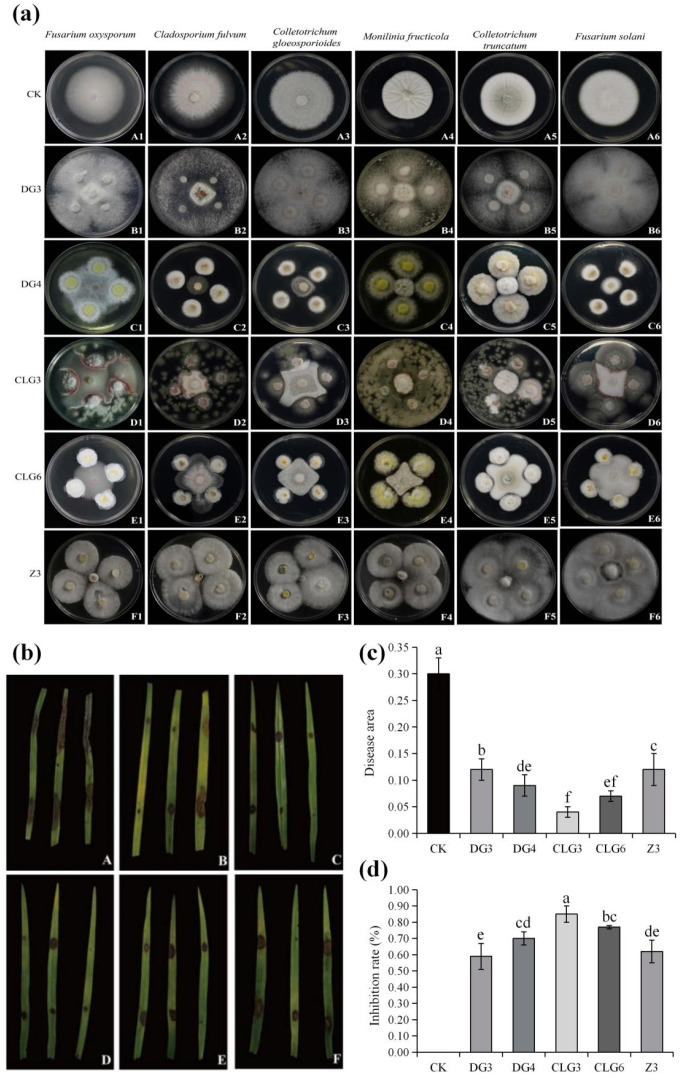
Antimicrobial effects of five endophytic fungal strains in vitro and in vivo. (**a**) Inhibitory effects of endophytic fungi against six pathogenic fungi in a plate assay. Pathogenic fungi were inoculated in the center of the plate, with endophytic fungi inoculated at four equidistant points. (**b**) Antifungal effects of five endophytic fungal strains against *F. oxysporum* (A: CK; B: DG3; C: DG4; D: CLG3; E: CLG6; F: Z3). (**c**) Disease area on plants following antagonism by endophytic fungi against *F. oxysporum*. (**d**) Inhibition rate of *F. oxysporum* growth by endophytic fungi. Data are expressed as mean ± standard deviation (SD) (*n* ≥ 3). Different lowercase letters indicate significant differences (*p* < 0.05) between different treatments.

The antimicrobial effects of five endophytic fungal strains in vitro were evaluated against several phytopathogens using a plate confrontation assay. All five strains inhibited pathogenic fungal growth to varying degrees (Figure 4a). Among them, strain Z3 displayed the strongest antagonistic activity, particularly against *Cladosporium fulvum* and *F. oxysporum*, with inhibition rates of 95.33% and 86.30%, respectively. Strains DG4 and DG3 also showed considerable activity, achieving inhibition rates greater than 70.14% against *F. oxysporum*, *Fusarium solani*, *Monilinia fructicola*, *Colletotrichum truncatum*, and *Cladosporium fulvum*. In contrast, strains CLG3 and CLG6 exhibited the weakest effects, with inhibition rates exceeding 73.01% only against *F. oxysporum* (Table 3).

The strains were co-cultured with tissue-cultured seedlings of *C. goeringii* for the in vivo experiment. Paraffin sections of the *C*. *goeringii* roots were stained with safranin O-fast green, and fungal infection status was observed under a 200× optical microscope. The results showed that all five fungal strains were able to colonize the root system of *C. goeringii* within 30 d. (Figure S2). After 30 d of symbiosis, there was a significant difference between the treatment group and the control group in antagonizing *F. oxysporum*. This was evidenced by smaller lesion areas on detached leaves from symbiotic seedlings, indicating enhanced resistance (Figure 4b,c). The calculated antimicrobial rates for all treatments exceeded 59% (Figure 4d). Overall, the antagonistic efficacy ranked as CLG3 > CLG6 > DG4 > Z3 > DG3.

### 3.4. The Growth-Promoting Effects of Five Endophytic Fungi on Seedlings of C. goeringii

We first tested the five endophytic fungi for pathogenicity and phytotoxicity. We inoculated tissue-cultured seedlings of *C. goeringii* with agar blocks containing these endophytic fungi and co-cultivated them for 30 d. The growth status of the seedlings was then observed, with seedlings inoculated with sterile culture medium blocks serving as the control group. After 30 d of co-cultivation, the treatment group showed corresponding increases in fresh weight, leaf number, and root number compared to the control group, as shown in Table S1. Morphologically, all five endophytic fungi were able to symbiotically associate with the *C. goeringii* seedlings, which remained upright and vibrant green (Figure S3). These results indicate that the five selected endophytic fungi are neither pathogenic nor phytotoxic, and can be used in subsequent experiments.

**Figure 5:**
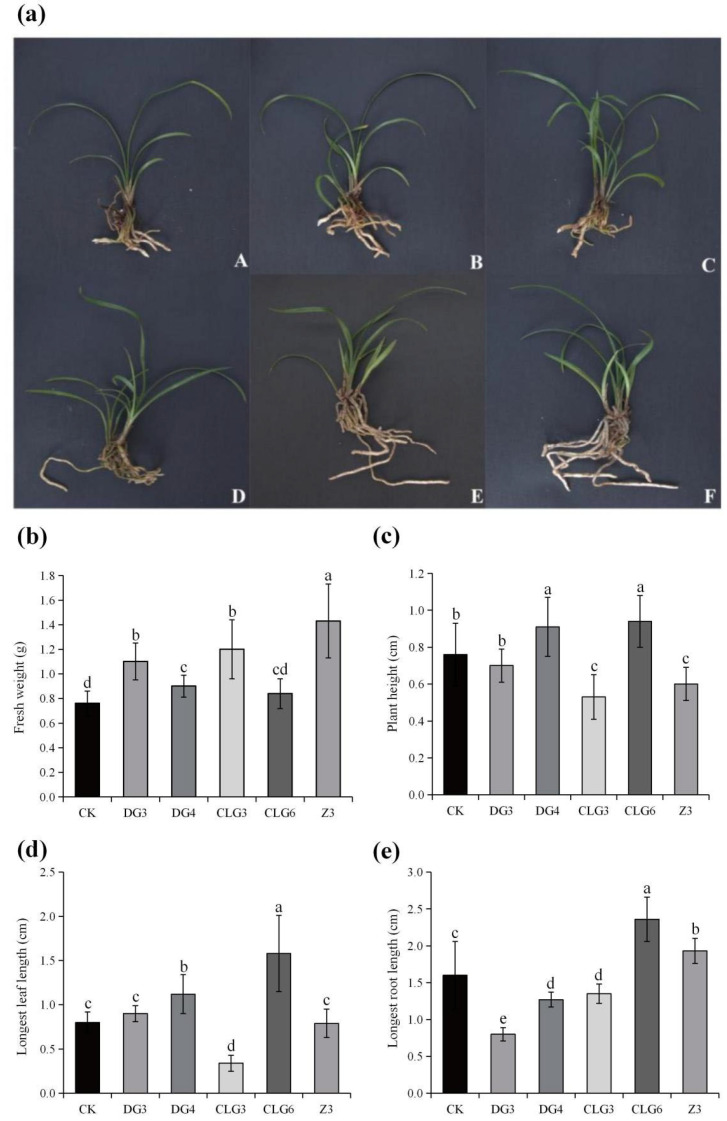
The growth-promoting effects of five endophytic fungi on seedlings of *C. goeringii*. (**a**): The growth condition of *C. goeringii* seedlings treated with the fermentation broth of endophytic fungi for 100 d (A: CK; B: DG3; C: DG4; D: CLG3; E: CLG6; F: Z3). The effect of endophytic fungi on *C. goeringii* seedlings fresh weight (**b**), plant height (**c**), longest leaf length (**d**), and longest root length (**e**). Data are expressed as mean ± standard deviation (SD) (*n* ≥ 3). Different lowercase letters indicate significant differences (*p* < 0.05) between different treatments.

Treatment of *C. goeringii* seedlings with endophytic fungal fermentation broth for 100 d significantly promoted growth compared to the control. This enhancement was evidenced by increased fresh weight and marked improvements in leaf length, leaf number, and root length (Figure 5a). The growth indicators of the symbiotic seedlings were measured. Among them, fresh weight increased in all treatment groups, with Z3 showing the highest promotion (88.16% above control), followed by CLG3 (57.89%), DG3 (44.74%), DG4 (18.42%), and CLG6 (10.53%) (Figure 5b). For plant height and longest leaf length, DG4 and CLG6 exhibited significant increases (Figure 5c,d). Regarding root growth, CLG6 also showed outstanding performance, leading in longest root length (47.5% longer) compared to the control (Figure 5e). In summary, while Z3 excelled in fresh weight, CLG6 demonstrated a broader growth-promoting advantage, leading in plant height, leaf length, and root length.

### 3.5. The Antagonistic Effects of Five Endophytic Fungi Against Stem Rot in C. goeringii Seedlings

The antagonistic effects of five endophytic fungi against stem rot in *C. goeringii* seedlings were analyzed. Compared to the control (water), seedlings treated with fungal fermentation broths (DG3, DG4, CLG3, CLG6, and Z3) exhibited significantly lower disease indices, with reductions of 43.01%, 29.18%, 36.53%, 64.19%, and 69.17%, respectively (Table 4). Among them, Z3 demonstrated the strongest biocontrol efficacy, achieving the lowest disease index and a control effect of 68.89%. These results indicate that Z3 not only promotes seedling growth but also effectively controls stem rot.

### 3.6. Effects of Five Endophytic Fungi on Physiological Indicators and Defense-Related Enzyme Activities in C. goeringii

**Figure 6:**
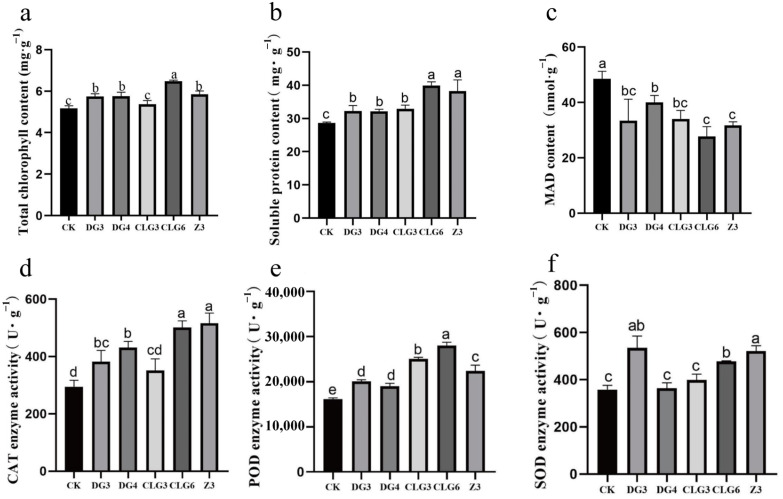
Physiological indicators and defense-related enzyme of *C. goeringii* seedlings treated with the fermentation broth of endophytic fungi for 100 d. (**a**): Total chlorophyll content; (**b**): Soluble protein content; (**c**): MDA content; (**d**): CAT enzyme activity; (**e**): POD enzyme activity; (**f**): SOD enzyme activity. Different lowercase letters indicate significant differences (*p* < 0.05) between different treatments.

Physiological indicators of the seedlings were measured to assess stress resistance. Compared to the control, chlorophyll content was significantly higher in DG3, DG4, CLG6, and Z3, with CLG6 showing the greatest increase (25.34%) (Figure 6a). Soluble protein content was elevated in all treatment groups, led by CLG6 39.54% increase and Z3 33.60% increase (Figure 6b). Conversely, MDA content was reduced across all treatments, suggesting improved membrane integrity (Figure 6c). Regarding antioxidant enzymes, Z3 exhibited the highest activities of SOD, CAT, and POD; DG3 and CLG6 also showed significantly higher activities of all three enzymes compared to the control (Figure 6d–f). These physiological enhancements indicate that treatment with fungal fermentation broths, particularly with strain Z3, DG3, and CLG6, bolstered the stress resistance of *C. goeringii*.

## 4. Discussion

### 4.1. Isolation and Identification of Endophytic Fungi from C. goeringii

Fungi are key symbiotic partners crucial for the growth and development of orchids, yet they also constitute a significant limiting factor in their artificial cultivation and domestication [23,24,25]. For instance, certain *Armillaria* species are essential symbionts of *Gastrodia elata*, as their rhizomorphs infect the plant’s nutritional stems and form digestible hyphae [26]. Similarly, a specific fungus (TU11) belonging to Tulasnellaceae (Basidiomycota) exhibits a highly specific association with *Dendrobium okinawense* [27]. In *Dendrobium wangliangii*, which is entirely dependent on mycorrhizal fungi for germination and growth, two *Fusarium* pro-germinating fungi have been identified [28]. Community-level studies further support this specialization. Xing et al. analyzed the orchid mycorrhizal fungal communities of eight *Dendrobium* species within a shared niche, revealing that phylogenetically related orchids tend to associate with similar sets of Tulasnellaceae fungi [29]. Functional studies demonstrate that the mycorrhizal fungus *Tulasnella calospora* actively supplies sulfur to the Mediterranean orchid *Serapias vomeracea* [30]. Finally, explorations of fungal communities in native habitats, such as those of *C. goeringii* and *Cymbidium faberi* in the Qinling region, have been conducted. This study isolated five endophytic fungi from the roots of *C. goeringii*. Based on morphological and molecular identification, they were classified as follows: DG3 as *Bjerkandera* sp. (Polyporaceae), DG4 as *Cylindrocarpon* sp. (Nectriaceae), CLG3 as *Talaromyces* sp. (Trichocomaceae), CLG6 as *Clonostachys* sp. (Nectriaceae), and Z3 as *Trichoderma* sp. (Hypocreaceae). Among them, *Talaromyces* sp., *Cylindrocarpon* sp., and *Clonostachys* sp. have been clearly confirmed as an endophytic fungus of orchids [31,32]. Although *Bjerkandera* sp., and *Trichoderma* sp. have been less frequently reported in orchids, they are widely distributed and functionally diverse in the plant kingdom as broad-spectrum, beneficial endophytic fungi [33,34].

Combined with previous research indicating that Ascomycota and Basidiomycota dominate the orchid rhizosphere and that geographic distribution, host species, and ecological type are key factors shaping mycorrhizal fungal diversity and specificity in *Cymbidium* [35], these results further demonstrate that orchid–fungal symbioses exhibit high specificity. This specificity is shaped by host plant species, nutritional mode, and geographic origin. Selecting appropriately matched fungal strains can therefore significantly enhance cultivation success rates. The pathogenicity and phytotoxicity of endophytic fungi are directly related to their potential for field application as biocontrol agents.

Although some species within the genus *Cylindrocarpon* are well-known plant pathogens, their biological functions exhibit significant strain specificity and host dependence. Recent studies have conclusively demonstrated that certain strains of this genus can exist as beneficial endophytic fungi. To determine whether the five selected endophytic fungi could be used on a large scale in the field, we analyzed their pathogenicity and phytotoxicity. The results showed that all tissue-cultured seedlings inoculated with the five endophytic fungi grew well, with upright and vibrant green leaves, and showed no signs of disease or root rot. Therefore, it is demonstrated that these five endophytic fungi are non-pathogenic and non-phytotoxic, and can be applied as biocontrol agents in the field.

### 4.2. Growth-Promoting of Endophytic Fungi from C. goeringii

Endophytes promote orchid growth and development by secreting plant hormones, contributing to photosynthesis, fixing nitrogen, enhancing mineral nutrient cycling, and producing siderophores and other bioactive compounds [36]. Four endophytic fungi *Colletotrichum gloeosporioides* (DJL-6), *Trichoderma tomentosum* (DJL-9), *Colletotrichum godetiae* (DJL-10), and *Talaromyces amestolkiae* (DJL-15) were isolated from *Cremastra appendiculata* tubers and screened for plant growth promotion. Experiments confirmed that these fungi possess strong nitrogen-fixing and potassium-solubilizing capabilities, significantly increasing the seed germination rate and plant biomass of *C. appendiculata* [37]. Nitrogen is an essential element for plant growth, closely linked to biomass accumulation and development [38]. Biological nitrogen fixation by fungi shows significant potential for promoting plant growth [39]. Endophytic diazotrophs, which colonize plant tissues and fix nitrogen in association with their hosts, can directly provide plants with this vital nutrient [40]. This is particularly relevant for orchids. Their tissues are highly nitrogen-enriched, and research indicates that orchids acquire nitrogen through symbiotic exchange with mycorrhizal fungi, a process reflected in the differential expression of genes related to nitrogen transport and metabolism in both partners [41]. Our study demonstrated that all five isolated endophytic fungal strains grew robustly on Ashby’s nitrogen-free medium, confirming their intrinsic nitrogen-fixing capacity (Figure 2c).

IAA, the most important endogenous auxin in plants, regulates nearly all aspects of plant growth and development. An appropriate concentration stimulates cell division, elongation, and adventitious root formation [42]. Endophytic fungi are known producers of IAA, which promotes host growth. For instance, in rice, an endophytic fungus regulates root IAA secretion to enhance nitrogen accumulation [43]; in *Glycyrrhiza glabra*, *Diaporthe terebinthifolli* GG3F6 optimizes IAA production, improving root/shoot growth and the accumulation of phenolics and flavonoids [44]. In orchids specifically, Shah et al. isolated 23 IAA-producing endophytes from *Dendrobium longicornu* and successfully amplified the *iaaM* gene from these fungi, confirming their biosynthetic pathway [45]. In our study, all five endophytic fungi from *C. goeringii* tested positive for IAA secretion in a colorimetric assay, with secretion capacity ranked as Z3 > DG3 > DG4 > CLG6 > CLG3 (deeper color indicates higher yield) (Figure 2a,b). Therefore, we hypothesize that these endophytic fungi promote the growth of *C. goeringii* through a combination of tolerance to nitrogen limitation and IAA secretion.

### 4.3. Biocontrol Potential of Endophytic Fungi from C. goeringii

Fungal endophytes represent a promising new source of biocontrol agents. For instance, endophytes such as *Clonostachys pseudochroleucha*, *Parathyridaria percutanea*, and *Curvularia lunata* have exhibited positive enzymatic activities, including amylase, lipase, protease, cellulase, and chitinase [46]. These enzymes can degrade key structural components of pathogens, contributing to biocontrol. In another example, a protease produced by the endophytic *Fusarium* strain JE-DP4a in papaya leaves was characterized as a neutral protease capable of catalyzing protein hydrolysis into smaller peptides or amino acids [47]. Furthermore, four endophytic fungal strains isolated from Amazonian plant leaves were shown to produce bioactive metabolites with antibacterial, antifungal, and antioxidant properties, and to enhance plant defense mechanisms [48]. Collectively, these studies illustrate the multifaceted potential of endophytic fungi in biological control. In the orchid *Cymbidium* sp., a qualitative plate screen identified several endophytic *Fusarium* species (*F. proliferatum*, *F. fujikuroi*, *F. incarnatum*, and *F. oxysporum*) with high L-asparaginase activity, supporting the view that orchid endophytes are producers of L-asparaginase and other bioactive compounds with diverse application potential [49]. In line with this, our qualitative analysis of biocontrol factors confirmed that all five tested endophytic fungal strains exhibited proteinase activity. As shown in Supplementary Figure S4, all five endophytic fungi in this study could produce transparent circles on skimmed milk medium, indicating that they all have the ability to produce protease. According to their D values, it can be known that DG3 > CLG6 > Z3 > CLG3 > DG4, and the activity level of DG3 is the highest (Table S2).

Iron is an essential plant micronutrient, playing a critical role in processes such as chlorophyll synthesis, photosynthesis, and respiration [50]. Endophytic fungi can enhance plant survival under iron-deficient conditions by upregulating root iron-related genes such as *FRO2* and *IRT1*, thereby increasing iron uptake and optimizing its distribution within the plant [51]. Furthermore, Li et al. reported that endophytic fungi can establish a stable iron metabolism network with their host plants, helping to prevent iron deposition and toxicity [52]. In this study, the qualitative results of the five endophytic fungi in this study are shown in Supplementary Figure S5. Only CLG3, CLG6, and Z3 presented transparent rings on the CAS medium. Therefore, it was determined that these 3 strains have the ability to produce siderophores, and the activity level is Z3 > CLG6 > CLG3 (Table S3). This finding suggests that their observed antimicrobial effects may involve competitive iron sequestration—depriving pathogens of this vital nutrient—and/or enhancing host plant vitality, thereby indirectly suppressing pathogen growth.

### 4.4. Antagonistic Effect Against F. oxysporum of Endophytic Fungi from C. goeringii

The interaction between plants and fungi is a complex process governed by numerous molecular factors that determine the nature of their mutualistic relationship [53]. For instance, in sugarcane, colonization by the nitrogen-fixing bacterium *Burkholderia* GXS16 was shown through RNA-seq and metabolomics to significantly activate root nutrient-uptake genes and promote root development [54]. Similarly, in *Dendrobium officinale*, the gene *DoSWEET14* plays a key role in regulating carbon allocation, thereby stabilizing the symbiotic relationship with its endophytic fungi [55]. In our study on *C. goeringii*, we observed a significant symbiotic-induced resistance. When challenged with *F. oxysporum*, detached leaves from seedlings that had formed a symbiotic association with our endophytic fungi showed significantly smaller lesion areas than non-symbiotic controls. All symbiotic treatments exhibited pathogen inhibition rates exceeding 59%. A comprehensive comparison ranked the antifungal efficacy of the strains as follows: CLG3 > CLG6 > DG4 > Z3 > DG3. However, the specific molecular mechanisms underlying this antagonism in *C. goeringii* endophytes require further elucidation through integrated transcriptomic and metabolomic analyses.

## 5. Conclusions

In this study, we isolated five endophytic fungi from the roots of *C. goeringii*: DG3 (*Bjerkandera* sp.), DG4 (*Cylindrocarpon* sp.), CLG3 (*Talaromyces* sp.), CLG6 (*Clonostachys* sp.), and Z3 (*Trichoderma* sp.). Our key findings are as follows. Regarding growth promotion, while Z3 excelled in fresh weight (88.16% above control), CLG6 demonstrated a broader growth-promoting advantage and led in plant height, leaf length, and root length. In terms of disease resistance, the five endophytic fungal strains exhibited stem rot-resistant effects. Z3 not only demonstrated the strongest antagonistic activity but also exhibited optimal preventive efficacy against stem rot disease with its fermentation broth, achieving a control effectiveness of up to 68.89%. Furthermore, treatment with fermentation broths from the five endophytic fungal strains resulted in increased total chlorophyll content and soluble protein content, decreased malondialdehyde content, and enhanced antioxidant enzyme activity in the leaves of *C. goeringii* seedlings. Collectively, these results demonstrate that all five endophytic fungi possess significant plant growth-promoting and broad-spectrum antagonistic potential in *C. goeringii*.

## Figures and Tables

**Table 1:** Classification standard for *C. goeringii* stalk rot.

Disease Index	Symptom
0	The entire plant shows no disease symptoms.
1	The disease occurs on the leaf sheaths of basal leaves, spreading from the leaf base toward the leaf tip.
2	25% or fewer leaves exhibit yellowing, and the base of the pseudobulb turns brown.
3	26–50% of the leaves exhibit yellowing, and the outer layer of the pseudobulb shows decay.
4	51–75% of the leaves exhibit yellowing, and the entire pseudobulb begins to decay.
5	More than 75% of the leaves exhibit yellowing, and the pseudobulb shows water-soaked soft rot.

**Table 2:** Biological morphological characteristics of five endophytic fungi.

Fungal Strain	Colony Morphology	Mycelial Morphology (40× Microscope)	Spore Morphology	Spore Dimensions
DG3	regular and circular in shape, with neat edges, a loose texture, and a white color	dendritic, with septa, the hyphae have clamp connections	short rod-shaped or elliptical, blunt at the apex, with a single septum occasionally observed	5–7 × 2–4 μm
DG4	regularly circular in shape, with neat edges, spaced concentric rings on the colony, dense texture, and pale yellow in color	branched, with septa	cylindrical in shape, with 1–3 septa	20–40 × 5–8 μm
CLG3	irregular in shape, dense in texture, and produces a reddish pigment with a cyan-brown hue	the terminal end produces broom-like conidiophores	elliptical in shape, aseptate	3–4 × 2–3 μm
CLG6	circular in shape, with neat edges, distinctly spaced concentric rings on the colony, dense texture, and a color ranging from pale yellow to orange-red	tree-like branching	elliptical, aseptate	9–12 × 3–4 μm
Z3	circular in shape, with neat edges, radiating pattern, loose texture, and white in color	phialides are produced at the apex of conidiophores	ovoid in shape, without septa	3–5 × 2–3 μm

**Table 3:** Inhibition rates (%) of endophytic fungi against 6 pathogenic fungi in dual culture.

Fungal Strain	*Fusarium oxysporum*	*Cladosporium fulvum*	*Colletotrichum gloeosporioides*	*Monilinia* *fructicola*	*Colletotrichum truncatum*	*Fusarium solani*
CK	0.00 ± 0.00 d	0.00 ± 0.00 e	0.00 ± 0.00 d	0.00 ± 0.00 e	0.00 ± 0.00 e	0.00 ± 0.00 d
DG3	78.64 ± 2.33 b	73.77 ± 0.87 b	68.33 ± 4.16 b	72.14 ± 0.58 c	74.03 ± 2.42 ab	71.38 ± 1.02 a
DG4	73.02 ± 0.25 c	70.14 ± 0.91 c	71.83 ± 1.61 b	75.57 ± 0.92 b	73.64 ± 1.78 b	75.00 ± 6.05 a
CLG3	73.01 ± 0.90 c	70.27 ± 0.38 c	68.67 ± 1.15 b	71.63 ± 0.83 c	62.79 ± 3.55 c	59.06 ± 3.49 b
CLG6	76.11 ± 1.00 bc	64.94 ± 0.89 d	58.33 ± 0.58 c	64.04 ± 1.28 d	55.04 ± 2.68 d	49.56 ± 2.72 c
Z3	86.30 ± 0.79 a	95.33 ± 1.53 a	83.87 ± 1.03 a	81.87 ± 0.32 a	78.67 ± 1.56 a	75.47 ± 0.57 a

Different lowercase letters indicate significant differences (*p* < 0.05) between different treatments.

**Table 4:** Control effect of stem rot disease in *C. goeringii* treated with fermentation broth of endophytic fungi.

Fungal Strain	Disease Index	Control Effect (%)
CK	60.52 ± 0.73 a	00.00 ± 00.00 e
DG3	34.49 ± 0.74 d	42.52 ± 1.24 c
DG4	42.86 ± 1.71 b	28.57 ± 2.85 e
CLG3	38.41 ± 0.86 c	35.98 ± 1.44 d
CLG6	21.67 ± 1.31 e	63.88 ± 2.19 b
Z3	18.66 ± 1.10 f	68.89 ± 1.83 a

Different lowercase letters indicate significant differences (*p* < 0.05) between different treatments.

## Data Availability

All data generated or analyzed during this study are included in this published article and are also available from the corresponding author upon reasonable request.

## Supplementary Materials

The following supporting information can be downloaded at: https://www.mdpi.com/article/10.3390/microorganisms14040758/s1, Figure S1: Disease severity scale diagram of stem rot in *C. goeringii*; Figure S2: Microscopic structure of *C. goeringii* roots colonized by endophytic fungi; Figure S3: Effects of strains of endophytic fungion the growth of tissue-cultured seedlings of *C. goeringii*; Figure S4: Status of endophytic fungi on skim milk agar plates; Figure S5: Status of endophytic fungi on CAS assay plates; Table S1: The effect of endophytic fungi on the growth of *C. goeringii* tissue culture seedlings; Table S2: Determination of proteinase activity levels of endophytic fungi; Table S3: Determination of siderophore production activity levels strains of endophytic fungi; Table S4: The base sequences of the five isolated endophytic fungal strains > CLG3.
