# Supplementary material for: Biocontrol Efficiency of Endophytic Fungi Against Stem-Rot in Cymbidium goeringii
Source: Microorganisms. 2026 Mar 27;14(4):758. doi: 10.3390/microorganisms14040758 (PMC13119494; doi:10.3390/microorganisms14040758)
Supplement: Supplementary file 1 [file microorganisms-14-00758-s001.zip › microorganisms-4176033-supplementary.pdf]

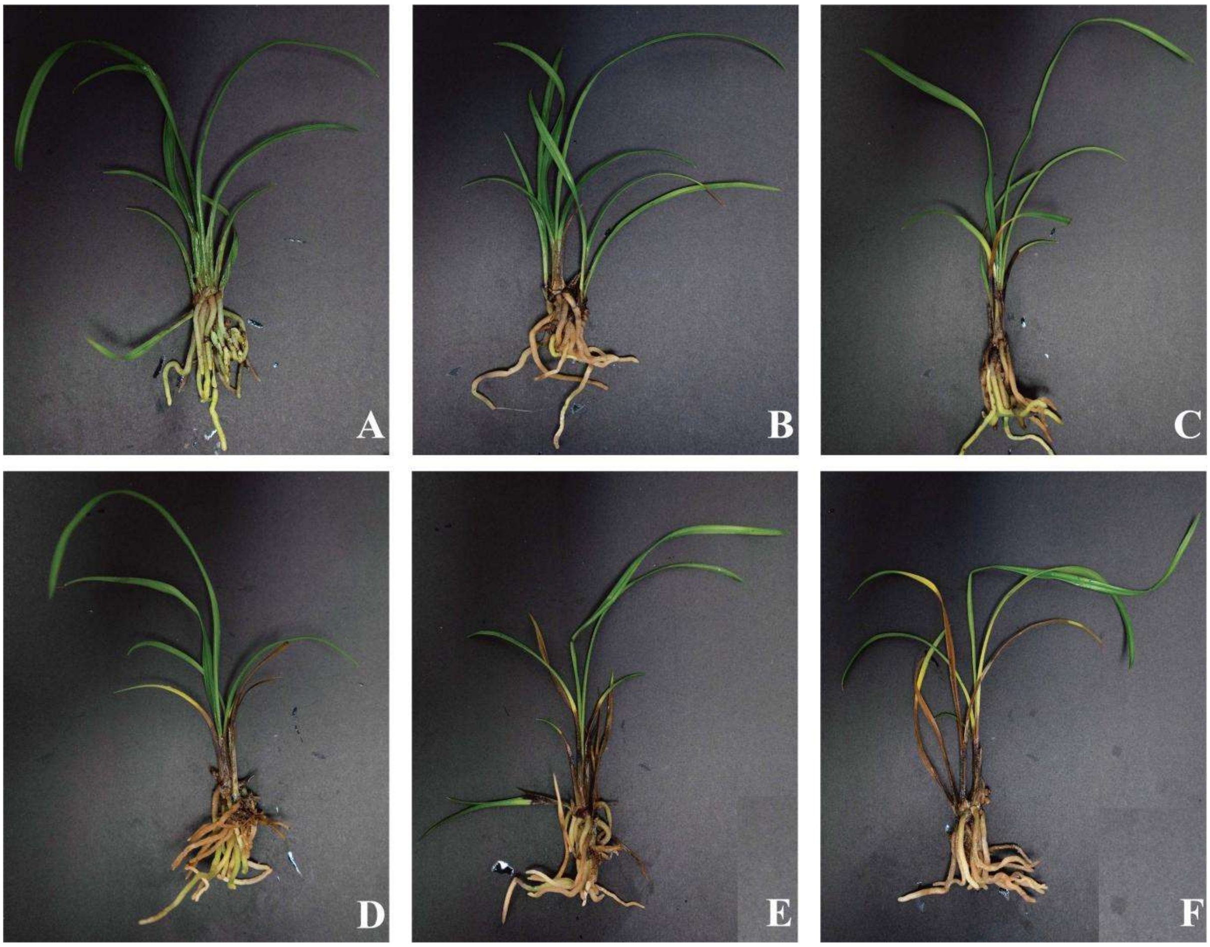

Supplementary Figure S1. Disease severity scale diagram of stem rot in *C. goeringii.* A: Grade 0; B: Grade 1; C: Grade 2; D: Grade 3; E: Grade 4; F: Grade 5.

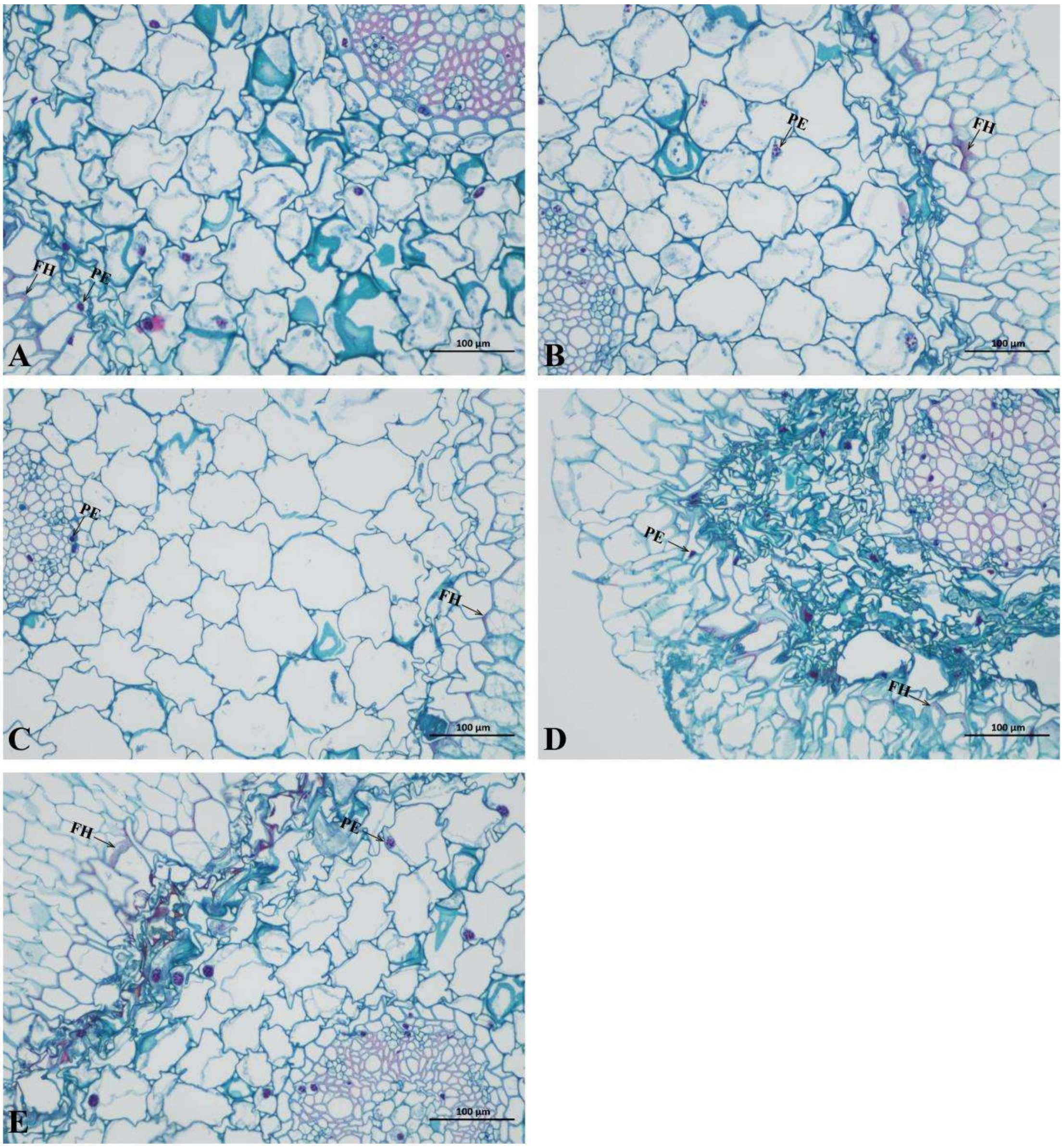

Supplementary Figure S2. Microscopic structure of *C. goeringii* roots colonized by endophytic fungi. A-E: Crosssections of *Cymbidium goeringii* roots colonized by endophytic fungi DG3, DG4, CLG3, CLG6 and Z3. PE: pelotons; FH: fungal hyphae.

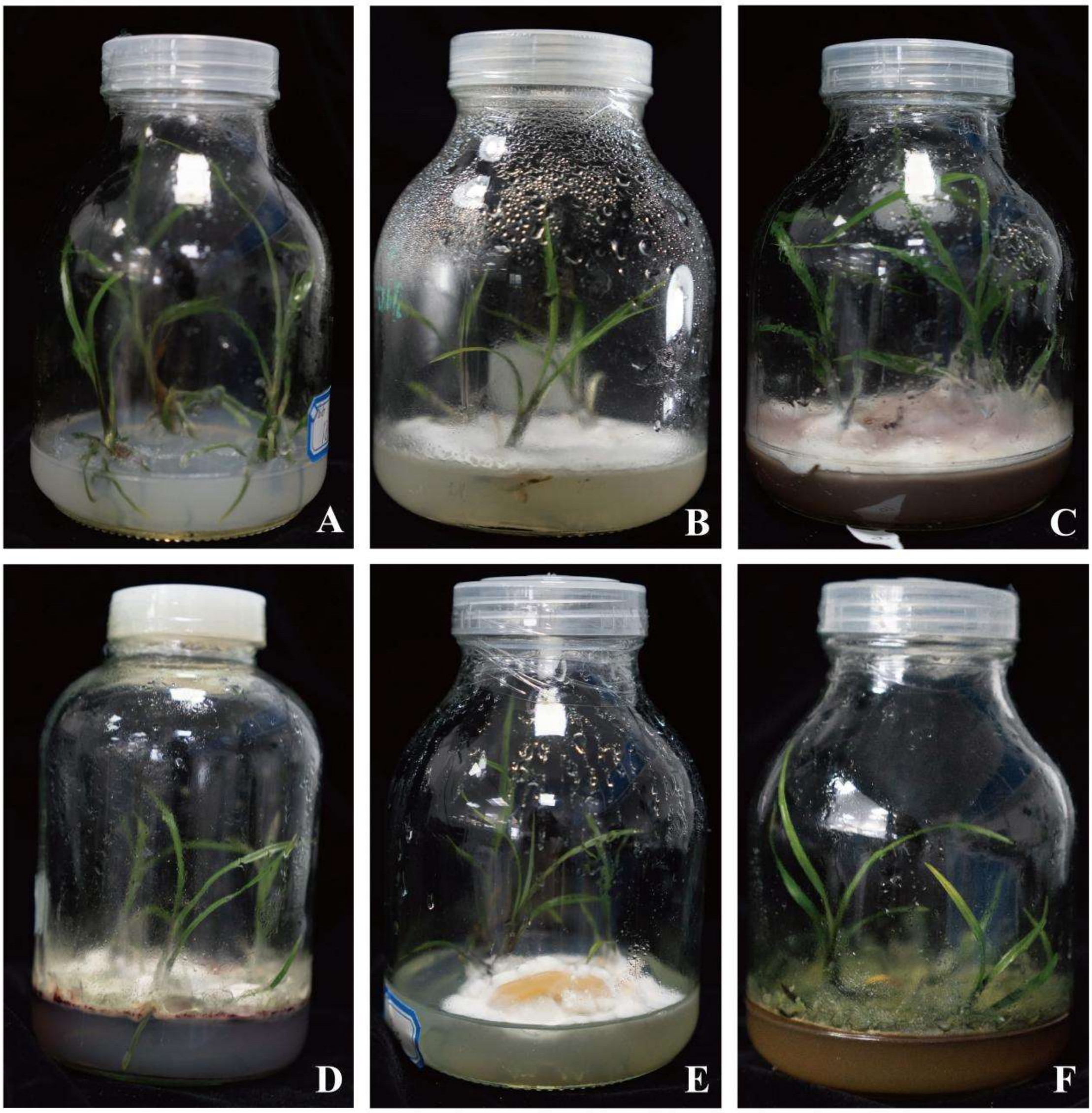

Supplementary Figure S3. Effects of strains of endophytic fungion the growth of tissue-cultured seedlings of *C. goeringii*. Note: A:CK;B:DG3;C:DG4;D:CLG3; E:CLG6;F:Z3

Supplementary Table S1. The effect of endophytic fungi on the growth of *C. goeringii* tissue culture seedlings.

Fungal Strain	Fresh Weight （g）	Plant Height (cm)	Longest Leaf Length (cm)	Number of Leaves (piece)	Number of Roots (pcs)
CK	1.09±0.12d	1.21±0.09d	0.97±0.15b	0.97±0.32b	0.77±0.43b
DG3	1.48±0.11a	1.66±0.14a	1.38±0.09a	0.90±0.31b	1.37±0.85a
DG4	1.20±0.09bc	1.49±0.09bc	0.67±0.11c	1.67±0.55a	0.73±0.45b
CLG3	1.26±0.10bc	1.56±0.10b	1.00±0.14b	0.77±0.63b	0.83±0.53b
CLG6	1.21±0.11c	1.02±0.85e	0.73±0.56c	1.03±0.56b	1.14±0.68ab
Z3	1.33±0.13b	1.42±0.17c	0.94±0.16b	0.83±0.53b	1.00±0.53ab

Note: The data in the table represent the increments of fresh weight, plant height, longest leaf length, leaf number, and root number after 30 d of symbiosis.

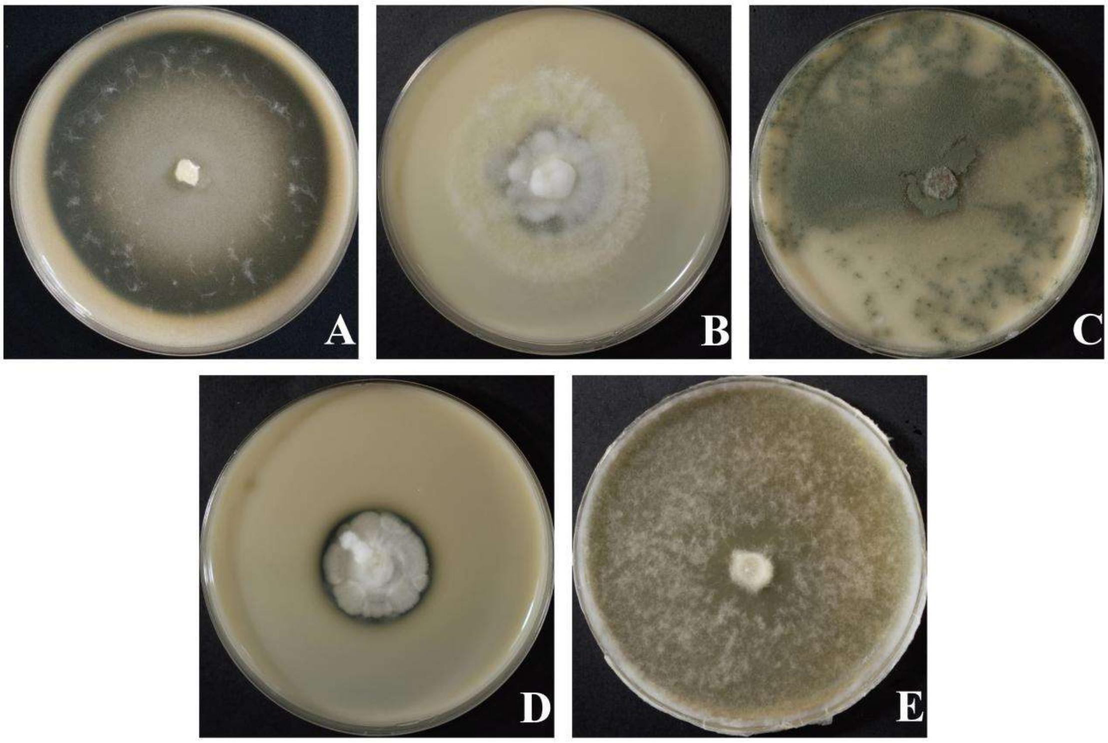

Supplementary Figure S4. Status of endophytic fungi on skim milk agar plates.
Note: A:CK;B:DG3;C:DG4;D:CLG3; E:CLG6;F:Z3

Supplementary Table S2. Determination of proteinase activity levels of endophytic fungi

Fungal Strain	Transparent circle diameter	Strain circle diameter	D-value	Activity level
DG3	7.40 ± 0.04b	5.48 ± 0.21b	1.92 ± 0.25a	+++
DG4	2.86 ± 0.13d	5.34 ± 0.28b	-2.48± 0.18d	+
CLG3	1.49 ± 0.03e	1.28 ± 0.13d	0.20± 0.11c	+
CLG6	3.03 ± 0.05c	2.63 ± 0.04c	0.39 ± 0.01b	+
Z3	8.90± 0.10a	8.67 ± 0.06a	0.23 ± 0.12c	+

Note: +++:D=1-2cm;++:D=0.5-1cm;+:D<0.5cm

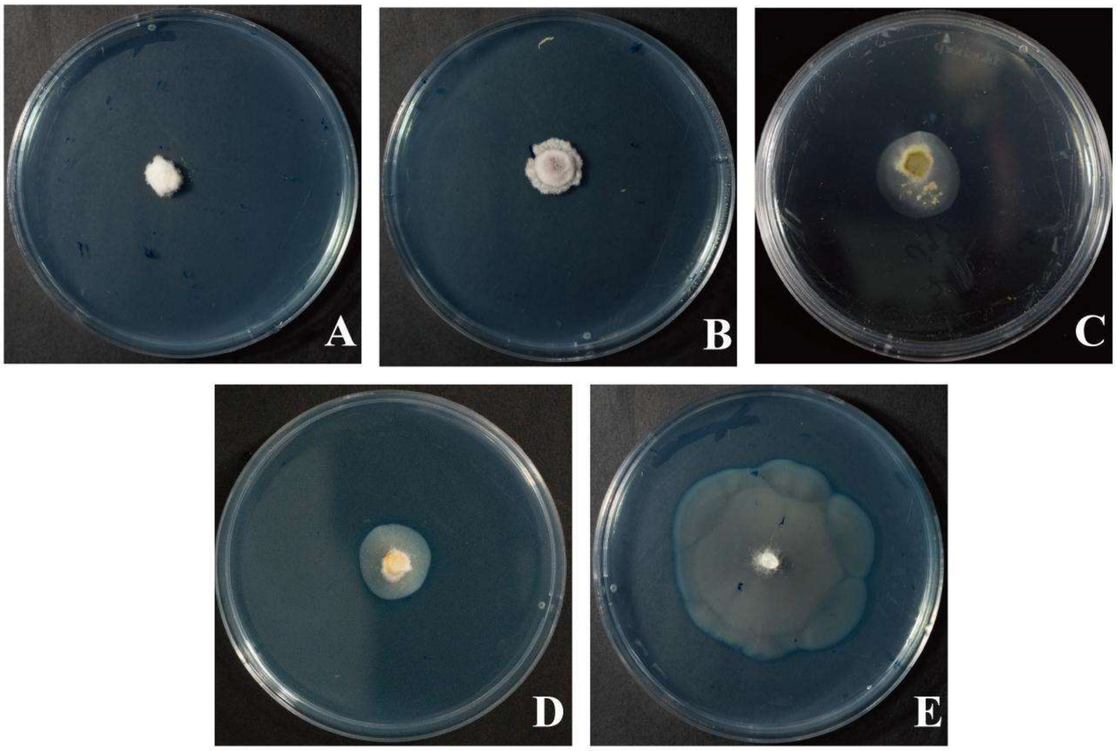

Supplementary Figure S5. Status of endophytic fungi on CAS assay plates

Note:A:CK;B:DG3;C:DG4;D:CLG3;E:CLG6;F:Z3

Supplementary Table S3. Determination of siderophore production activity levels strains of endophytic fungi

Fungal Strain	Transparent circle diameter	Strain circle diameter	D-value	Activity level
CLG3	2.25±0.22b	1.76±0.17a	0.49±0.06c	+
CLG6	1.95±0.09c	0.76±0.02c	1.19±0.11b	+++
Z3	5.47±0.44a	0.90±0.12b	4.57±0.33a	++++

Note:++++:D>2cm;+++:D=1-2cm;++:D=0.5-1cm;+:D<0.5cm

Supplementary Table S4. The base sequences of the five isolated endophytic fungal strains

>CLG3

CCTTCCGTTAGGGGGGCCTGCGGAAGGATCATTACCGAGTGCGCGGCCCCTCGTGGCC
CAACCTCCCACCCTTGTCTCTATACACCTGTTGCTTTGGCGGGCCCACCGGGGCCACCT
GGTCGCCGGGGGACGTTCGTCCCCGGGCCCGCGCCCGCCGAAGCGCTCTGTGAACCC
TGATGAAGATGGGCTGTCTGAGTACTATGAAAATTGTCAAAACTTTCAACAATGGATCT
CTTGGTTCCGGCATCGATGAAGAACGCAGCGAAATGCGATAAGTAATGTGAATTGCAG
AATTCCGTGAATCATCGAATCTTTGAACGCACATTGCGCCCCCTGGCATTCCGGGGGGC
ATGCCTGTCCGAGCGTCATTTCTGCCCTCAAGCACGGCTTGTGTGTTGGGTGCGGTCCC
CCCGGGGGCCTGCCCGAAAGGCAGCGGCGACGTCCGTCTGGTCCTCGAGCGTATGGG
GCTTTGTCACTCGCTCGGGAAGGACTGGCGGGGGTTGGTCACCACCACAAAATTTTAC
CACGGTTGACCTCGGATCAGGTTAGGAGTTACCCGCTGAACTTAAGCATATCAAAAGG

CGGGAGGAATG

>CLG6

GGGGGGCCTGCGGAGGGATCAGTACCGAGTTTACAACTCCCAAACCCATGTGAACATA
CCTATCGTTGCTTCGGCGGGATCGCCCCGGGTGCCTTGCGTGCCCCGGATCCAGGCGC
CCGTCAAAGGACCTTAACTCTTGTATTATTTTGGAATCTTCTGAGTCGTTTTTACAAATA
AATAAAAACTTTCAACAACGGATCTCTTGGTTCTGGCATCGATGAAGAACGCAGCGAA
ATGCGAAAAGTAATGTGAATTGCAGAATTCAGTGAATCATCGAATCTTTGAACGCACAT
TGCGCCCGCCAGTATTCTGGCGGGCATGCCTGTCTGAGCGTCATTTCAACCCTCATGCC
CCTAGGGCGTGGTGTTGGGGATCGGCCAAGGCCCGCAAGGGACGGCCGGCCCCTAAA
TCTAGTGGCGGACCCGTCGTGGCCTCCCCTGCGAAGTAGTGATATTCCGCATCGGATAG
CGACGAGCCCCTGCCGTTAAACCCCCCAAACTTTACAAGGTTGACCTCAGATCAGGTA
GGAATACCCGCTGAACTTAAGCATATCAAAAGGCGGGAGGAA

>DG3

CTTCCGTTAAGGGGACCTGCGGAAGGATCATTATCGAGTTTTGAATGGGGTTGTCTGCT
GGCTCGCAAGGGCATGTGCACGCCTGTCTCATCCACTCTCAACTTCTGTGCACTTTTCA
TAGGCCGGCTTGTGGGTGCGTTCGCGCACTTGTAGGTGTCGGGCTTATGCTTTACTACA
AACGATTCAGTTTTAGAATGTCATACTTTGCTATAACGCAATTATATACAACTTTCAGCA
ACGGATCTCTTGGCTCTCGCATCGATGAAGAACGCAGCGAAATGCGATAAGTAATGTG
AATTGCAGAATTCAGTGAATCATCGAATCTTTGAACGCACCTTGCGCTCCTTGGTATTC
CGAGGAGCATGCCTGTTTGAGTCTCATGGAATTCTCAACCTTCAGCTTTATTGATGAAG
GCTTGGACTTGGAGGTCGTGCCGGCTCTCGTAGTCGGCTCCTCTGAAATGCATTAGTGC
GAACGTTACCAGCCGCTTCAGCGTGATAATTATCTGCGTTGCTGTGGAGGGTATTCTAG
TGTTCACGCTTCTAACCGTCTTCGGACAAATTTCTGAACTCTGAGCTCAAATCAGGTAG
AACTACCCGCTGAACTTAAGCATATCAAAAGCCGGAGGAA

> DG4

TTCCGTATGAGGGGGGCCTGCGGAGGGATCATTACCGACGGAACAACTCCCAAACCCC
TGTGAACATACCAAACGTTGCTTCGGCGGACCACCCCGACCCCTTCGCGGGCGACGGG
CCCGCCAGAGGACACCCAAAATTCAAATGTATTTTGTTTTCAAAACGTATTCTGAGTGG
AATTTTTAAATAAATCAAAACTTTCAACAACGGATCTCTTGGCTCTGGCATCGATGAAG
AACGCAGCGAAATGCGATAAGTAATGTGAATTGCAGAATTCAGTGAATCATCGAATCTT
TGAACGCACATTGCGCCCGCCAGTATTCTGGCGGGCATGCCTGTTCGAGCGTCATTTCA
ACCCTCAAGCCCCCGGGCTTGGTGTTGGGGATCGGCATCAGGCGCTGGCACCTCCGGG
AGCCGCGCCCCGCCGTCCCTCAAATACAGTGGCGGTCACGCCGTGTGTACCTCAGCGT
AGTAGCAACACCTCGCTCCGGGACCCTTCTGTGACCACGCCGTAAAACCCCCGACTTT
TTTCTGGTTGACCTCGAATCAGGAAGGACTACCCGCTGAACTTAAGCATATCAAAAATC
GGGAGGAAA

>Z3

CCTGCGGAGGGATCATTACCGACTCCACAACACCCAAACCCAATGTGAACGTTACCAA
ACTGTTGCCTCGGCGGGATCTCTGCCCCGGGTGCGTCGCAGCCCCGGACCAAGGCGCC
CGCCGGAGGACCAACCAAAACTCTTTTTGTATACCCCCTCGCGGGTTTTTTATAATCTG
AGCCTTCTCGGCGCCTCTCGTAGGCGTTTCGAAAATGAATCAAAACTTTCAACAACGG
ATCTCTTGGTTCTGGCATCGATGAAGAACGCAGCGAAATGCGATAAGTAATGTGAATTG
CAGAATTCAGTGAATCATCGAATCTTTGAACGCACATTGCGCCCGCCAGTATTCTGGCG
GGCATGCCTGTCCGAGCGTCATTTCAACCCTCGAACCCCTCCGGGGGGTCGGCGTTGG

GGATCGGCCCTGCCTCTTGGCGGTGGCCGTCTCCGAAATACAGTGGCGGTCTCGCCGC
AGCCTCTCCTGCGCAGTAGTTTGCACACTCGCATCGGGAGCGCGGCGCGTCCACAGCC
GTTAAACACCCAACTTCTGAAATGTTGACCTCGGATCAGGTAGGAATACCCGCTGAAC
TTAAGCATATCAA
